# Prescribing Patterns of Dupilumab for Atopic Dermatitis in Adults: Retrospective, Observational Cohort Study

**DOI:** 10.2196/41194

**Published:** 2023-08-30

**Authors:** Torunn E Sivesind, Ani Oganesyan, Grace Bosma, Camille Hochheimer, Lisa M Schilling, Robert Dellavalle

**Affiliations:** 1 Department of Dermatology University of Colorado School of Medicine Aurora, CO United States; 2 University of Colorado School of Medicine Aurora, CO United States; 3 Center for Innovative Design and Analysis The Colorado School of Public Health University of Colorado School of Medicine Aurora, CO United States; 4 Department of Medicine University of Colorado School of Medicine Aurora, CO United States; 5 Division of General Internal Medicine University of Colorado School of Medicine Aurora, CO United States; 6 The Colorado School of Public Health University of Colorado School of Medicine Aurora, CO United States; 7 Dermatology Service Eastern Colorado Health Care System US Department of Veterans Affairs Denver, CO United States

**Keywords:** dupilumab, atopic dermatitis, systemic treatment, biologics, monoclonal antibody, prescribing patterns, dermatitis, adults, disease, immune response, data, inflammatory, immune, dermatology, dermatologist, eczema, Dupixent, asthma, nasal polyp, chronic sinusitis, eosinophilic, neurodermatitis

## Abstract

**Background:**

Atopic dermatitis (AD) is a common inflammatory disease caused by a type 2 T helper cell–mediated immune response to environmental antigens. Approximately 1 in 5 patients with AD presents with moderate to severe disease, and treatments approved by the Food and Drug Administration include emollients, topical glucocorticoids, and calcineurin inhibitors. Dupilumab, a fully human monoclonal antibody, improves AD via inhibition of interleukin-4 and interleukin-13.

**Objective:**

Our aim was to characterize the prescribing patterns of dupilumab for AD in adults at a large university-affiliated health system.

**Methods:**

A retrospective, observational cohort study was conducted using electronic data from the Observational Health Data Sciences and Informatics database, assessing data from the University of Colorado Medical Campus and its affiliates. The outcome measured was the prevalence of dupilumab prescribed for adults with AD (n=6421), between March 28, 2013, and March 28, 2021. We assessed whether the characteristics of patients who received dupilumab were different from those who did not. Each patient characteristic was assessed using a univariate logistic regression with the binary outcome of receiving or not receiving dupilumab.

**Results:**

We found a population prevalence of 5.6% (6421/114,476) for AD. In our cohort, Black patients with AD were more than twice as likely to have received dupilumab compared to White patients (odds ratio 2.352, 95% CI 1.58-3.39). Patients with a diagnosis of atopic neurodermatitis were approximately twice as likely to have received dupilumab compared to those with other diagnostic variants of AD (odds ratio 1.87, 95% CI 1.01-3.22).

**Conclusions:**

Our results demonstrate that both patient racial characteristics and specific AD diagnoses were associated with variations in dupilumab prescription patterns.

## Introduction

Atopic dermatitis (AD) is a chronic inflammatory disease that affects US children and adults, with a reported prevalence of 10%-13% and 7%, respectively [1-4]. Higher disease severity is associated with lower quality of life, worsened mental status, and higher use of health care resources [1,5]. This includes emergency department visits and hospitalizations as well as increased pharmaceutical and outpatient costs [6-8]. Consequently, effectively treating patients with AD has substantial clinical and economic implications.

Assessing the severity of AD is determined by the number of sites involved (eg, head and neck, upper extremities, or trunk and lower extremities), the lesion characteristics (eg, erythema, edema or papulation, and lichenification), as well as reported symptoms. Patients who have 10%-29% bodily involvement with notable signs of inflammation are classified as having moderate disease, and those with greater or equal to 30% bodily involvement are considered to have severe disease. This classification is important in the diagnosis and treatment of AD, thereby affecting therapeutic outcomes [9]. Treatment of AD with a systemic immunomodulating agent is indicated when the disease is considered moderate to severe [10]. A variety of systemic agents, including cyclosporine, azathioprine, methotrexate, mycophenolate mofetil, and systemic steroids, are used in practice without strict guidelines or recommendations to guide treatment choices [10,11]. In March 2017, dupilumab became the first biologic drug approved for the treatment of AD in adults, and in May 2020, it gained approval for use in children aged 6 years and older. Dupilumab blocks the interleukin (IL)-4 alpha receptor, inhibiting IL-4 and IL-13 signaling and preventing the release of type 2 cytokines that promote inflammation in AD [12]. Compared with the aforementioned systemic immunomodulating agents, dupilumab may be more effective as a long-term maintenance therapy and has the advantage of an overall improved side-effect profile, with no required drug-specific laboratory monitoring [11]. However, access to this immunomodulator may be limited by its novelty and cost (depending upon the dose, up to US $59,000/year for patients without insurance) [13].The disease burden of AD disproportionately affects non-Hispanic Black patients; the source of this disparity is multifactorial. Although the specific gene-environment interactions in the pathophysiology of AD are unknown, many factors—such as differences in environmental pollution, contact with tobacco smoke, hygiene practices, access to health care, diet, and exposure to disease—likely play a role [4,14,15]. Loss of function mutations in the filaggrin gene is linked with an increased risk of developing AD, oftentimes leading to persistent disease. Filaggrin loss-of-function mutations are less common in Black patients compared to White patients, yet Black patients are still more likely to experience persistent disease [16,17]. In addition, Black patients have lower skin ceramide/cholesterol ratios, attenuated T helper 1 and T helper 17 immunophenotypes, and higher serum immunoglobulin (Ig) E levels, which predisposes to skin breakdown, dysregulated immunity, and increased inflammation [18,19]. Despite higher AD disease severity and increased health care needs, Black patients are less likely to receive outpatient dermatologic care [3,20-22]. A prior investigation reporting on race- and ethnicity-related disparities in the treatment of AD found that Black patients had statistically significant lower odds of receiving dupilumab compared to White patients [23]. Previous research has also shown that Black patients with psoriasis are less likely than White patients to receive biologic treatment, independent of demographic or socioeconomic factors and comorbidities [24,25].

Data related to the prescription patterns of dupilumab for AD are needed to inform health equity and decision-making in everyday practice.

## Methods

### Ethical Considerations

The Colorado Multiple Institutional Review Board determined that this research did not involve human subjects, and therefore, was exempt from ethics approval.

### Data Source

We performed a retrospective, observational cohort study of adult patients treated for AD, using electronic data from the University of Colorado Anschutz Medical Campus and its affiliates (hospital wards and outpatient clinics) via Health Data Compass (HDC), an electronic health data warehouse [26]. Records of patients who visited any of the institution’s facilities from January 1, 2010, to March 28, 2021, were pulled from HDC and remained in the format of the Observational Health Data Sciences and Informatics (OHDSI) Observational Medical Outcomes Partnership common data model. Data extracted included demographics, prescription history, diagnosis history, and visit details.

### Study Design and Study Population

**Figure 1:**
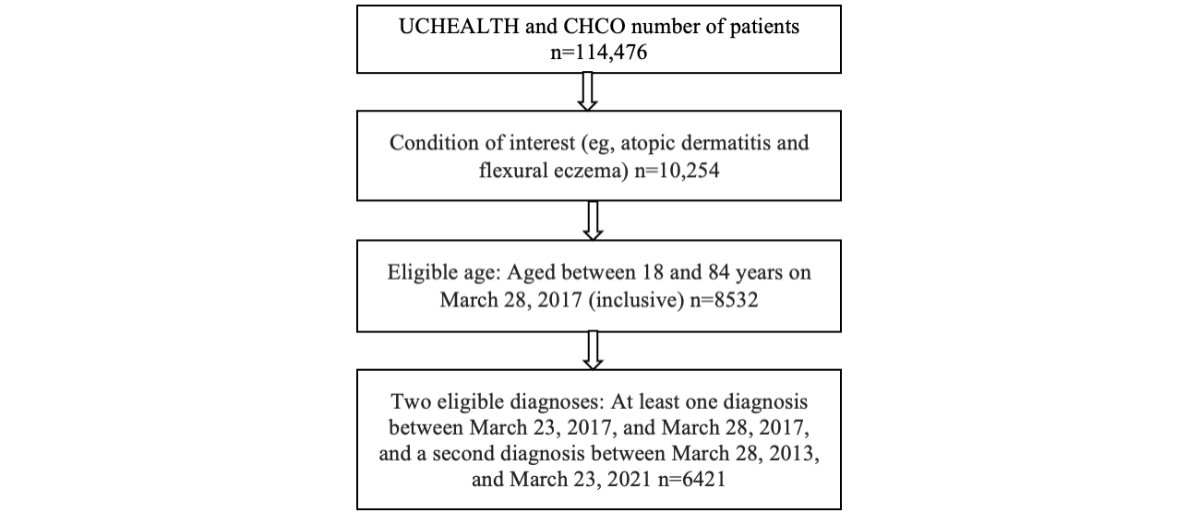
Flowchart of included patients. UCHEALTH: University of Colorado Health; CHCO: Children's Hospital Colorado.

The outcome of interest was the prevalence of dupilumab prescribed for AD, stratified by patient characteristics. The study cohort, consisting of individuals with specific AD diagnoses and various dupilumab prescription types, was developed using the OHDSI Atlas cohort and concept set tools (Table 1). All study participants were between the ages of 18 and 85 years as of March 28, 2017, with a diagnosis of AD, as defined by at least two encounter diagnoses of AD (Figure 1). The drug of interest was any prescription order of dupilumab (200 mg or 300 mg syringe or pen). Records included were those of ordered medications. Information regarding fulfilment for this study was not used, nor did we include orders external to our institution. Included dupilumab prescriptions were required to have a start date on or after March 28, 2017, and to have occurred following a diagnosis of AD. Prescriber data, including their medical credential and practice setting, were not analyzed.

**Table 1:** Concept set for diagnosis of atopic dermatitis, displaying *International Classification of Diseases, Tenth Revision* (*ICD-10*) source codes that map to the included Observational Medical Outcomes Partnership concept IDs.

Concept ID	Concept code	Concept name	Class	Domain	Vocabulary
45601213	L20.84	Intrinsic (allergic) eczema	5-character billing code	Condition	*ICD-10-CM^a^*
45596150	H60.54	Acute eczematoid otitis externa	5-character nonbilling code	Condition	*ICD-10-CM*
45581716	H60.549	Acute eczematoid otitis externa, unspecified ear	6-character billing code	Condition	*ICD-10-CM*
45572269	L30.1	Dyshidrosis (pompholyx)	*ICD-10* code	Condition	*ICD-10-CM*
45572263	L20.83	Infantile (acute, chronic) eczema	5-character billing code	Condition	*ICD-10-CM*
45567351	L20.89	Other atopic dermatitis	5-character billing code	Condition	*ICD-10-CM*
45562535	L30.0	Nummular dermatitis	*ICD-10* code	Condition	*ICD-10-CM*
45557698	L20	Atopic dermatitis	*ICD-10* hierarchy	Condition	*ICD-10-CM*
45557486	H60.542	Acute eczematoid otitis externa, left ear	6-character billing code	Condition	*ICD-10-CM*
45557485	H60.541	Acute eczematoid otitis externa, right ear	6-character billing code	Condition	*ICD-10-CM*
45552974	L20.82	Flexural eczema	5-character billing code	Condition	*ICD-10-CM*
45548191	L20.9	Atopic dermatitis, unspecified	*ICD-10* code	Condition	*ICD-10-CM*
45547951	H60.543	Acute eczematoid otitis externa, bilateral	6-character billing code	Condition	*ICD-10-CM*
45533637	L20.8	Other atopic dermatitis	*ICD-10* code	Condition	*ICD-10-CM*
35208498	L30.1	Dyshidrosis (pompholyx)	4-character billing code	Condition	*ICD-10-CM*
35208497	L30.0	Nummular dermatitis	4-character billing code	Condition	*ICD-10-CM*
35208450	L20.9	Atopic dermatitis, unspecified	4-character billing code	Condition	*ICD-10-CM*
1569766	L20.8	Other atopic dermatitis	4-character nonbilling code	Condition	*ICD-10-CM*
1569765	L20	Atopic dermatitis	3-character nonbilling code	Condition	*ICD-10-CM*
1418386	L30.100	Dyshidrosis (pompholyx)	*ICD-10* code	Condition	*ICD-10-CM*
1418385	L30.1	Dyshidrosis (pompholyx)	*ICD-10* code	Condition	*ICD-10-CM*
1418384	L30.000	Nummular dermatitis	*ICD-10* code	Condition	*ICD-10-CM*
1418383	L30.0	Nummular dermatitis	*ICD-10* code	Condition	*ICD-10-CM*
1418239	L20.900	Atopic dermatitis, unspecified	*ICD-10* code	Condition	*ICD-10-CM*
1418238	L20.9	Atopic dermatitis, unspecified	*ICD-10* code	Condition	*ICD-10-CM*
1418237	L20.806	Newborn skin eczema (machine translation)	*ICD-10* code	Condition	*ICD-10-CM*
1418236	L20.805	Diffuse neurodermatitis (machine translation)	*ICD-10* code	Condition	*ICD-10-CM*
1418235	L20.804	Baby eczema (machine translation)	*ICD-10* code	Condition	*ICD-10-CM*
1418234	L20.803	Atopic neurodermatitis (machine translation)	*ICD-10* code	Condition	*ICD-10-CM*
1418233	L20.802	Allergic eczema (machine translation)	*ICD-10* code	Condition	*ICD-10-CM*
1418232	L20.801	Neurodermatitis (machine translation)	*ICD-10* code	Condition	*ICD-10-CM*
1418231	L20.800	Other atopic dermatitis	*ICD-10* code	Condition	*ICD-10-CM*
1418230	L20.8	Other atopic dermatitis	*ICD-10* code	Condition	*ICD-10-CM*
1418227	L20	Atopic dermatitis	*ICD-10* hierarchy	Condition	*ICD-10-CM*

^a^ *ICD-10-CM: International Classification of Diseases, Tenth Revision, Clinical Modification.*

### Statistical Analysis

We assessed whether the characteristics of patients with AD who received dupilumab were different from those who did not. Each patient characteristic was gathered based on AD-associated billing and nonbilling codes. These codes were based on the *International Classification of Diseases, Tenth Revision* and are listed in Table 1. The characteristics were then assessed using a univariate logistic regression, with the binary outcome of receiving dupilumab or not receiving dupilumab (Table 2). The resulting *P* values associated with multilevel categorical characteristics (eg, race and diagnosis) were corrected for multiple testing using the False Discovery Rate (Benjamini-Hochberg correction) method. Reference levels included White race, non-Hispanic ethnicity, female sex, and AD as the first eligible diagnosis.

**Table 2:** Summary statistics—overall and by patients who did and did not receive dupilumab.

User prevalence		Did not receive (n=6172)	Received (n=249)	Overall (N=6421)
Age (years), mean (SD)		53.3 (17.9)	51.4 (16.7)	53.2 (17.9)
Age (years), median (min, max)		54.0 (22.0, 89.0)	52.0 (22.0, 87.0)	54.0 (22.0, 89.0)
**Sex, n (%)**				
	Female	3532 (57.2)	146 (58.6)	3678 (57.3)
	Male	2621 (42.5)	102 (41)	2723 (42.4)
	Missing	19 (0.3)	1 (0.4)	20 (0.3)
**Race, n (%)**				
	American Indian and Alaska Native	20 (0.3)	1 (0.4)	21 (0.3)
	Asian	246 (4)	11 (4.4)	257 (4)
	Black or African American	397 (6.4)	34 (13.7)	431 (6.7)
	Multiple race	161 (2.6)	6 (2.4)	167 (2.6)
	Native Hawaiian and other Pacific Islander	12 (0.2)	0 (0)	12 (0.2)
	White or Caucasian	4912 (79.6)	179 (71.9)	5091 (79.3)
	Other	340 (5.5)	13 (5.2)	353 (5.5)
	Missing	84 (1.4)	5 (2.0)	89 (1.4)
**Ethnicity, n (%)**				
	Hispanic	563 (9.1)	20 (8)	583 (9.1)
	Non-Hispanic	5491 (89)	226 (90.8)	5717 (89)
	Missing	118 (1.9)	3 (1.2)	121 (1.9)
**Diagnosis, n (%)**				
	Atopic dermatitis	3050 (49.4)	213 (85.5)	3263 (50.8)
	Atopic neurodermatitis	107 (1.7)	14 (5.6)	121 (1.9)
	Flexural eczema	758 (12.3)	5 (2)	763 (11.9)
	Nummular eczema	1120 (18.1)	6 (2.4)	1126 (17.5)
	Vesicular eczema	1137 (18.4)	11 (4.4)	1148 (17.9)

## Results

Summary statistics gathered based on the *International Classification of Diseases, Tenth Revision* billing codes are provided in Table 2. There were 249 dupilumab prescriptions among 6421 patients. Our cohort had a mean age of 53.2 (SD 17.9) years and was composed of mostly non-Hispanic (n=5491, 89%), White (n=4912, 79.3%), and female (n=3532, 57%) patients. The most common recent diagnosis was a general diagnosis of atopic dermatitis (n=3263, 50.8%), followed by vesicular eczema (n=1148, 17.9%), nummular eczema (n=1126, 17.5%), flexural eczema (n=763, 11.9%), and lastly, atopic neurodermatitis (n=12, 1.9%). Among those who received dupilumab, the mean age was 51.4 (SD 16.7) years and 58.6% (n=146) were female. Every patient who received a prescription had either multiple prescriptions, prescriptions for quantities greater than 1, or received refills. The majority of patients were White (n=179, 71.9%), followed by patients identifying as Black or African American (n=34, 13.7%) and “Other” races (n=13, 5.2%). Among the most common recent diagnoses, 85.8% (n=213) were general diagnoses of AD, and the remaining were diagnoses of atopic neurodermatitis (n=14, 5.6%), flexural eczema (n=5, 2.2%), nummular eczema (n=6, 2.2%), or vesicular eczema (n=11, 4.1%).

We assessed whether the proportion of patients who received dupilumab was different based on patient characteristics. Each patient characteristic (Table 3) was assessed using logistic regression with the binary outcome of receiving dupilumab or not receiving it. The *P* values associated with multilevel categorical characteristics (eg, race or diagnoses) were corrected for multiple testing using the False Discovery Rate (Benjamini-Hochberg correction) method. Reference levels included white race, non-Hispanic ethnicity, female sex, and AD as the first eligible diagnosis.

In our cohort, Black patients were approximately twice as likely to have received dupilumab for AD compared to White patients (odds ratio 2.352, 95% CI 1.58-3.39). Similarly, those diagnosed with atopic neurodermatitis were about twice as likely to have received dupilumab compared to those who were diagnosed with AD (odds ratio 1.87, 95% CI 1.01-3.22). Conversely, those with other eczema diagnoses, including flexural eczema, nummular eczema, or vesicular eczema, were less likely to have received dupilumab compared to those with a most recent diagnosis of atopic neurodermatitis.

**Table 3:** Univariate analysis by patient characteristics.

Variable		Odds ratio (CI)	*P* value
Age (per 10-year increase)		0.944 (0.879-1.013)	.11
**Sex**			
	Male	1.00 (reference)	—^a^
	Female	0.941 (0.726-1.217)	.65
**Race**			
	American Indian or Alaska Native	—	—
	Asian	1.228 (0.622-2.183)	.86
	Black or African American	2.352 (1.583-3.397)	<.001
	Multiple Race	1.024 (0.398-2.15)	.96
	Native Hawaiian or Other Pacific Islander	—	—
	White or Caucasian	1.00 (reference)	—
	Other	1.05 (0.564-1.791)	.96
**Ethnicity**			
	Non-Hispanic or Latino	1.00 (reference)	—
	Hispanic or Latino	0.863 (0.526-1.340)	.54
**Diagnoses**			
	Atopic dermatitis	1.00 (reference)	—
	Atopic neurodermatitis	1.87 (1.01-3.22)	.03
	Flexural eczema	0.0945 (0.0335-0.207)	<.001
	Nummular eczema	0.0767 (0.0302-0.158)	<.001
	Vesicular eczema	0.139 (0.0708-0.243)	<.001

^a^Not applicable.

## Discussion

### Principal Findings

We found that patient racial characteristics as well as specific eczema diagnoses were associated with different frequencies of dupilumab prescriptions.

Specifically, our results suggest that patients who reported their race as African American or Black were more likely to have received a dupilumab prescription compared to White patients in our health care system. This result is surprising, as previous research demonstrated less outpatient dermatologic care and fewer biologic prescriptions among the population of Black patients with AD [27,28].

Our study also revealed that patients with a diagnosis of atopic neurodermatitis were twice as likely to be prescribed dupilumab compared to patients with a diagnosis of AD. The term “neurodermatitis” refers to localized, circumscribed patches of lichenified skin that are commonly associated with itching. This term is increasingly being used to describe skin eruptions associated with anxiety [29].

Our study also revealed that patients with specific AD diagnoses, such as flexural eczema, nummular eczema, and vesicular eczema, are less likely to receive dupilumab than those with general AD, without further specification. One possible explanation may be that patients with certain subtypes of AD respond well to more conservative, first-line therapies (ie, topical corticosteroids, emollients, and topical calcineurin inhibitors) [30].

### Limitations

Although the study results provide novel insights pertaining to the prescribing practices of dupilumab, there are important limitations. Data capturing patients’ insurance status and type of insurance at the time of visit would have been useful to provide further clarity regarding prescribing relationships; unfortunately, our HDC data warehouse only has the current insurance status and type available from our electronic health record system. Furthermore, the type of health care provider prescribing dupilumab as well as their specific sector of medical employment were not analyzed. Additional studies evaluating the background of the clinicians prescribing dupilumab may provide a better understanding of these unique prescription patterns.

### Conclusions

We found that Black patients and patients diagnosed with atopic neurodermatitis were approximately twice as likely to have received dupilumab compared to White patients and those with a diagnosis of AD, respectively. Future studies may consider exploring possible factors contributing to the dupilumab prescription patterns we discovered, such as AD disease severity as a potential contributing factor and the role of insurance type in dupilumab prescribing frequency. Investigating similar trends among other OHDSI network sites might also be beneficial in elucidating the trends noted among our study population and would contribute to the generalizability of the results. Furthermore, there is little existing data regarding the treatment of AD in the pediatric demographic—additional research would allow clinicians to provide evidence-based care to this population subset.

## Abbreviations

AD: atopic dermatitis
HDC: Health Data Compass
IL: interleukin
OHDSI: Observational Health Data Sciences and Informatics
